# Ensemble deep-learning networks for automated osteoarthritis grading in knee X-ray images

**DOI:** 10.1038/s41598-023-50210-4

**Published:** 2023-12-21

**Authors:** Sun-Woo Pi, Byoung-Dai Lee, Mu Sook Lee, Hae Jeong Lee

**Affiliations:** 1 Division of AI and Computer Engineering, Kyonggi University, Suwon, Republic of Korea; 2 Department of Radiology, Keimyung University Dongsan Hospital, Daegu, Republic of Korea; 3 Department of Pediatrics, Samsung Changwon Hospital, Sungkyunkwan University School of Medicine, Changwon, Republic of Korea

**Keywords:** Musculoskeletal system, Information technology, Computational science

## Abstract

The Kellgren–Lawrence (KL) grading system is a scoring system for classifying the severity of knee osteoarthritis using X-ray images, and it is the standard X-ray-based grading system for diagnosing knee osteoarthritis. However, KL grading depends on the clinician’s subjective assessment. Moreover, the accuracy varies significantly depending on the clinician’s experience and can be particularly low. Therefore, in this study, we developed an ensemble network that can predict a consistent and accurate KL grade for knee osteoarthritis severity using a deep learning approach. We trained individual models on knee X-ray datasets using the most suitable image size for each model in an ensemble network rather than using datasets with a single image size. We then built the ensemble network using these models to overcome the instability of single models and further improve accuracy. We conducted various experiments using a dataset of 8260 images from the Osteoarthritis Initiative open dataset. The proposed ensemble network exhibited the best performance, achieving an accuracy of 76.93% and an F1-score of 0.7665. The Grad-CAM visualization technique was used to further evaluate the focus of the model. The results demonstrated that the proposed ensemble network outperforms existing techniques that have performed well in KL grade classification. Moreover, the proposed model focuses on the joint space around the knee to extract the imaging features required for KL grade classification, revealing its high potential for diagnosing knee osteoarthritis.

## Introduction

Osteoarthritis (OA), also known as degenerative arthritis, is a common joint disease that causes pain, deformity, and dysfunction in the bones, joint membranes, and surrounding ligaments that form joints due to gradual damage or degenerative changes in the articular cartilage. Knee OA is one of the leading causes of disability in the elderly and is the most common form of OA^1^. The pain caused by knee OA has a serious impact on patient quality of life, and the fact that there is currently no effective treatment to inhibit the degenerative structural changes responsible for the progression of knee OA leads to even more serious consequences for patients^2^. Currently, the only treatments available to patients with knee OA to temporarily relieve pain and slow down the progression of OA are weight loss and appropriate joint muscle strengthening exercises^3^. Therefore, early detection of knee OA can further improve the patient’s quality of life.

**Figure 1:**
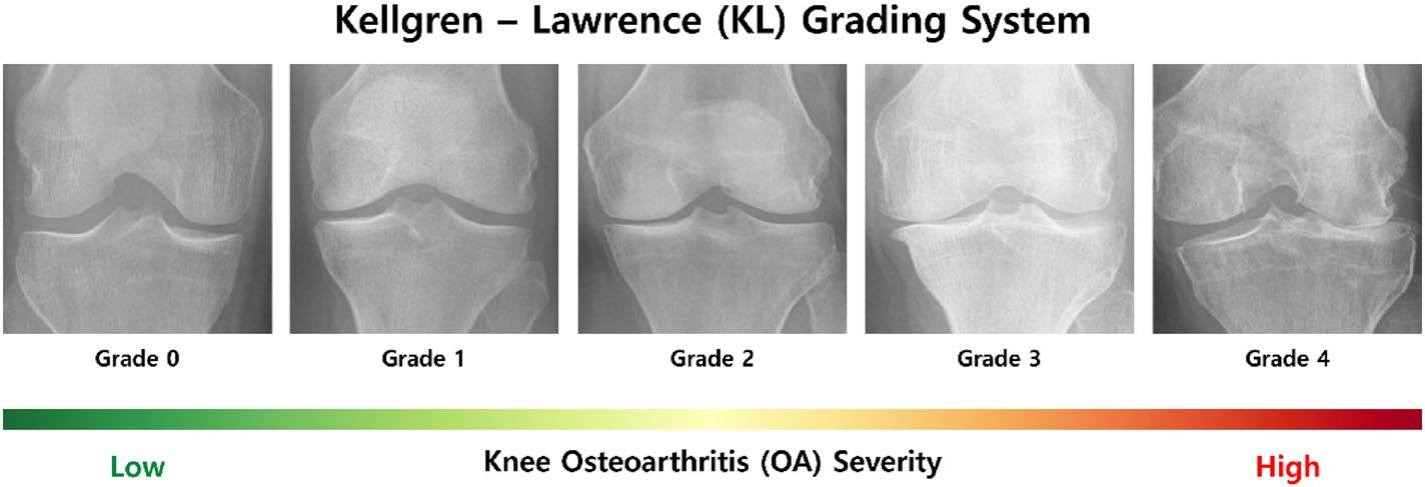
Examples of knee joint X-ray images for each KL grade according to OA severity. The KL grade system categorizes grades according to two crucial aspects of OA: the extent of joint space narrowing and the degree of osteophyte formation.

There are several methods for diagnosing OA, such as X-ray examination, arthroscopic examination, and MRI examination. X-ray examination is widely available and affordable; hence, it is the most commonly used method for diagnosing OA. The standard method for measuring the severity of knee OA using X-ray images is the Kellgren–Lawrence (KL) grading system, which uses five grades ranging from 0 to 4 according to severity^4^. Figure 1 shows example knee X-ray images for each KL grade according to knee OA severity. It is evident that joint space narrowing (JSN), osteophyte, and severe bone deformity occur as the knee OA severity progresses from grade 0 to grade 4.

However, assessing the severity of knee OA and assigning a KL grade using X-ray images depends on the clinician who is determining the KL grade. Hence, the accuracy of the assessment varies significantly depending on the clinician’s experience and can be inaccurate. To lessen the dependency on clinician subjectivity and reduce the cost of diagnosis using X-ray images, an automated system based on a deep learning approach is needed^5^. The performance of image classification in deep learning has improved substantially because of increased computing power, large datasets that have been made available to the public, and the development of convolutional neural networks (CNNs). Moreover, deep learning-based image classification methods have surpassed the conventional computer vision techniques and can be applied to the KL grading of knee OA in medical imaging^6^. Unlike conventional machine learning approaches, deep learning extracts and learns features from data on its own without human intervention. Hence, it is possible to make a more objective, consistent, and accurate diagnosis of knee OA using an automated measurement system that is based on deep learning^7^.

We developed an automated KL grade classification model that classifies the severity of knee OA and assigns a KL grade. The model employs a deep learning approach based on CNNs. The idea behind the approach used in this study is that the optimal image sizes for training different deep learning models are also variable. We used different image sizes to train each model and conducted experiments to demonstrate that an ensemble network composed of these trained models performs very well in determining the severity of knee OA. In addition, we verified the superiority of the individual deep learning models and the ensemble network they form using the Grad-CAM visualization technique.

## Related work

Shamir et al.^8^ proposed a weighted nearest-neighbor algorithm that integrates techniques such as texture features, Gabor filters, and Chebyshev statistics to predict KL grade. However, as the performance of image classification based on deep learning has increased significantly in recent years, many such methods have been developed, along with several attempts to automatically predict KL grades using deep learning. Antony et al.^9^ approached the KL grade prediction problem as a regression problem rather than a classification problem and used transfer learning with pre-trained CNNs along with the mean-squared loss as the loss function. In their later study, Antony et al.^10^ used a weighted combination of cross-entropy loss and mean-squared error loss to train CNNs from scratch. They obtained satisfactory results that outperformed existing approaches. Chen et al.^2^ proposed a new ordinal loss to replace the existing cross-entropy loss to fine-tune a network for KL grade classification. They reported that better performance was achieved using the ordinal rather than the cross-entropy loss function. Moreover, Yong et al.^11^ proposed an ordinal regression module (ORM) for a neural network to formulate the KL grade prediction as a regression problem. They optimized the network using cumulative-link loss and achieved significant improvement in KL grade prediction. Tiulpin et al.^12^ proposed a new KL grade prediction method based on a deep Siamese CNN^13^, which is a network that learns the similarity metrics between image pairs to determine knee OA severity. Furthermore, Tiulpin et al.^14^ later used two independent datasets, the Osteoarthritis Initiative (OAI) dataset^15^ and the Multicenter Osteoarthritis Study (MOST) dataset^16^, to predict the KL grade considering overall knee OA severity. They also predicted the Osteoarthritis Research Society International (OARSI) grade, which considers the characteristics of individual parts of the knee. They used an average ensemble of the two models to improve performance for KL grade and OARSI grade prediction. Jain et al.^17^ proposed OsteoHRNet, which integrates the convolutional block attention module (CBAM)^18^ based on HRNet^19^, a high-resolution network that enables efficient classification considering the spatial scale of images. In addition, they optimized the proposed network for the KL grade classification problem using ordinal loss.

## Materials and methods

This retrospective study received approval from the Institutional Review Board of Kyonggi University, where the research was conducted (IRB No. KGU-20230216-HR-098). All methods adhere to the ethical standards outlined in the Helsinki Declaration. The Institutional Review Board of Kyonggi University waived the need for informed consent because the data used in this retrospective study were already fully de-identified to protect patient confidentiality.

### Dataset

In this study, we used the knee X-ray images provided by Chen et al.^2,20^, which were created from the OAI open dataset. The OAI dataset is publicly available, and it was obtained from a 10-year observational study of knee OA severity. The KL grades of the OAI dataset were determined by three musculoskeletal radiologists. The dataset provided by Chen et al. consists of 8260 posterior-anterior (PA) fixed flexion X-ray images of the left and right knees. These images were generated from a total of 4796 participants including both male and female patients aged 45 to 79 years. The original dataset consists of 5778 images in the training set, 826 images in the validation set, and 1656 images in the test set, following a ratio of 7:1:2. This dataset exhibits class imbalance among different KL grades. However, previous studies reported that addressing the class imbalance issue using various sampling techniques, including oversampling, undersampling, and weighted sampling, did not provide meaningful performance improvement^2,11,14,17^. Therefore, in this study, we employed the stratified fivefold cross-validation technique^21^ across a dataset of 6,604 images, which combines the 5,778 images from the training set and the 826 images from the validation set. Additionally, we maintained a 4:1 ratio of training to validation data for each KL grade. We note that the testing dataset remained entirely separate and was not involved in the training process. This methodology ensures that the label distribution remains consistent in both the training and validation datasets, thus mitigating issues related to class imbalance. Table 1 provides an overview of the dataset composition of the dataset we used.

**Table 1:** Dataset composition. In this study, we employed a five-fold cross-validation technique on 6,604 images, comprising 5,778 images for the training set and 826 images for the validation set during the training process. The test set was kept entirely separate and was not utilized in the training process.

Dataset	Total number of images	KL grade	Number of images
Training Set (training & validation)	6604 (5283 & 1321)	0	2614
		1	1199
		2	1728
		3	863
		4	200
Test Set	1656	0	639
		1	296
		2	447
		3	223
		4	51

### Network architecture

This section presents the details of the ensemble network as well as the deep learning models used in this study. In general, better performance can be achieved when an ensemble technique is used because the final output stems from a combination of the predictions of multiple classification models^22^. Therefore, in this study, an ensemble network consisting of several representative image classification models, DenseNet-161^23^, EfficientNet-b5^24^, EfficientNet-V2-s^25^, RegNet-Y-8GF^26^, ResNet-101^27^, ResNext-50-32×4d^28^, Wide-ResNet-50-2^29^, and ShuffleNet-V2-×2-0^30^, was used to predict KL grades. The models used in this study are best-known as models for feature extraction and classification tasks for natural images, and their efficiency has been demonstrated^31^. In this study, we used pre-trained models because they predicted KL grades better when the weights of each model were pre-trained on a large dataset like ImageNet than when they were randomly initialized. The final layer of each model was modified to output the five KL grades (0 to 4). Subsequently, the output values for these five grades are passed through a softmax non-linear function^32^ and then transformed into probability values representing each KL grade for the given image. The output value for the KL grade of each model and the probability value corresponding to that output are then provided as inputs to the final prediction layer to predict the final KL grade.

For the ensemble learning, we applied a technique called *mix voting*, which addresses the limitations of hard voting by using hard and soft voting to predict the final KL grade. In the hard voting method, the grade predicted by the highest number of models is selected as the final KL grade for a given image. However, the hard voting method is limited because it cannot derive the final KL grade when there are two or more KL grades that have been predicted by the same number of models. For example, if four out of eight models predict a KL grade of 0 while the remaining four models predict a KL grade of 1, two KL grades have been predicted by the highest number of models. By contrast, the soft voting method takes the arithmetic average of the probability values representing the KL grades predicted by each model and determines the grade with the highest probability value to be the final KL grade.

**Figure 2:**
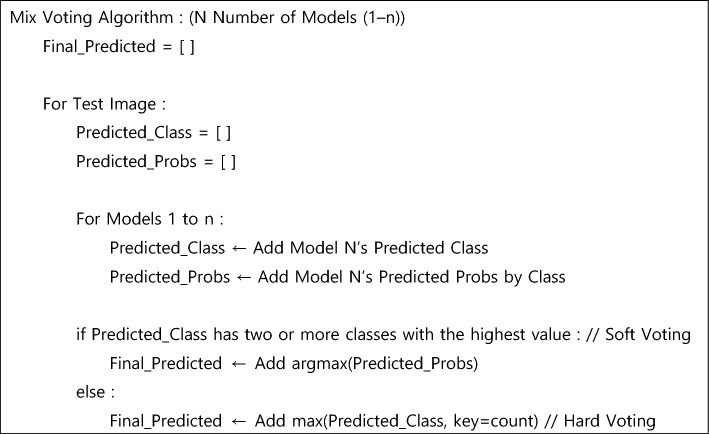
Mix voting algorithm pseudo code—Mix voting essentially follows the hard voting approach. However, in cases where two or more grades receive an equal and the highest number of votes, the algorithm utilizes the soft voting method to determine the final KL grade.

The mix voting method is similar to the hard voting method in that it selects the KL grade predicted by the highest number of models as the final KL grade. However, if there are two or more KL grades that have been predicted by the same number of models, the mix voting method employs the soft voting method to derive the final KL grade. Figure 2 presents the algorithm for the mix voting ensemble method.

### Data preprocessing and augmentation

According to previous studies, the size of the images used to train and test a deep learning model can affect its classification performance. Moreover, the model’s performance can be enhanced significantly by using the optimal image size^33^. Therefore, in this study, to select the optimal image size for each deep learning-based classification model comprising the ensemble network, the models were trained using twelve candidate image sizes. The candidate images were divided into two groups. The first group consisted of square-sized images where the width and height were the same, and the other group was composed of non-square images. Then, the KL grade classification performance of the models was evaluated according to the image size.

The candidate square image sizes included the original image size of 224 × 224 pixels as well as sizes of 300 × 300 pixels, 384 × 384 pixels, 448 × 448 pixels, 456 × 456 pixels, and 512 × 512 pixels. For non-square images, to prevent significant distortion when resizing the original image, we set the aspect ratio of the target image to not exceed 1:2 between its shorter and longer sides. As a result, we used sizes of 224 × 336 pixels, 224 × 448 pixels, 300 × 450 pixels, 336 × 224 pixels, 448 × 224 pixels, and 450 × 300 pixels. We constructed datasets with these twelve image sizes and then conducted our experiments. When resizing the images, we used the cubic convolution interpolation with 4 × 4 neighbor pixels. Among the datasets resized to different image sizes, the training image set was augmented with several versions of the images during the training process. In this case, at each epoch, an image was dynamically transformed into an augmented image and employed for training. Specifically, each image was randomly flipped horizontally with a 50% probability or rotated by a random angle between − 20° and + 20° with a 50% probability during each epoch of the training process. Consequently, for a given source image, a minimum of one and a maximum of three images derived from the same source were utilized in the training process. Once each model was trained, the test time augmentation (TTA) technique was applied to the model to predict KL grades. TTA is a type of data augmentation ensemble method. This technique augments the images in the test set while evaluating the trained model to improve performance without additional training^34^. In the TTA, horizontal flip augmentation was applied to each test image. Each model predicted the KL grade using a total of two images for each test image: the original test image and the horizontally flipped test image.

**Figure 3:**
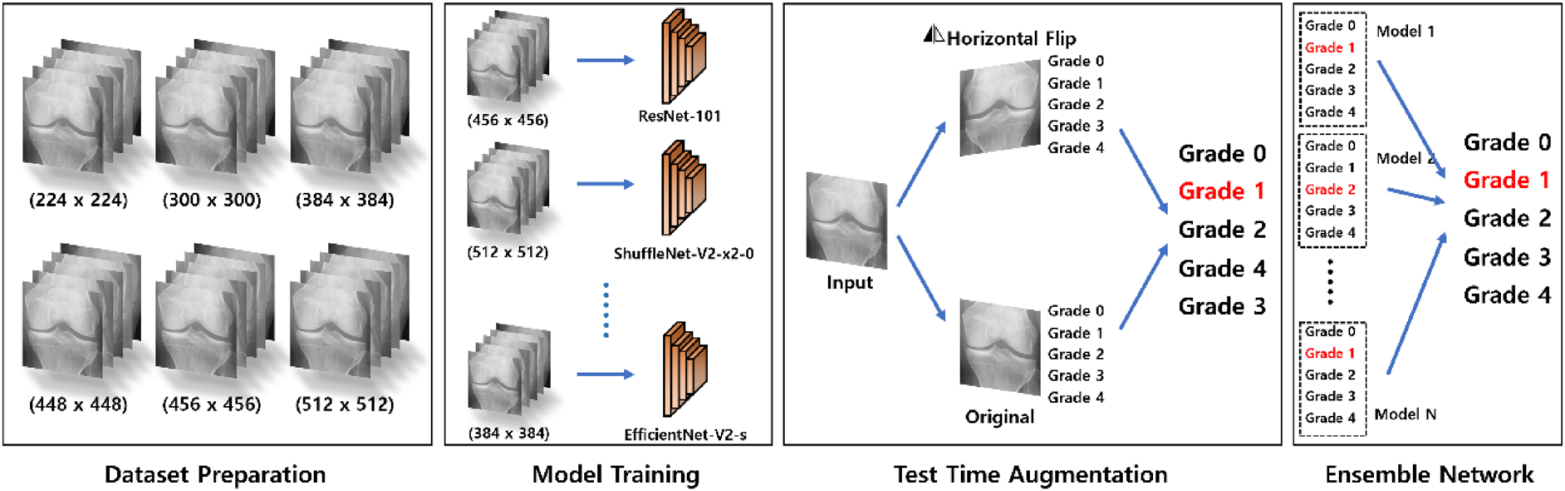
Pipeline of the model for classifying the KL grade for knee OA severity. After selecting the optimal image size out of the six candidate sizes, the models were trained with images of the selected optimal size. An ensemble network was then constructed using the trained models to predict the final KL grade.

In addition to image resizing and data augmentation, we also tried histogram normalization, histogram equalization, contrast limited adaptive histogram equalization (CLAHE), and preprocessing that horizontally flips the left knees in the entire dataset to make them all oriented on the right side. However, none of these approaches resulted in significant performance improvement, and in some cases the performance even decreased. Hence, we did not include them in the reported results of this study. Figure 3 shows the pipeline of the model used to classify an image according to the KL grade for knee OA severity.

### Training strategy

The Python programming language (version 3.10) and the PyTorch deep learning framework (version 1.12.1) were employed for all model training and experiments in this study. All experiments were conducted using an NVIDIA A100 GPU with a 40GB memory capacity. For training efficiency and quicker convergence, this study considered batch sizes that are powers of 2, and we empirically determined the batch size to be 16, considering the GPU memory size utilized during training.

Optimizing hyperparameters for deep learning models can be challenging due to the high dimensionality of the search space. In this study, we adopted the training strategy introduced by Tiulpin et al.^14^ for eight different models pre-trained on ImageNet, subsequently retraining each model for images of different-sized KL grades. To elaborate, during the initial epoch, we froze all layers except for the Fully Connected (FC) layer responsible for classification and trained the model with a learning rate of 0.01. In the subsequent epoch, we unfroze all layers, decreased the learning rate to 0.001, and continued the training process. Finally, we further reduced the learning rate to 0.0001 and continued training for the remaining epochs. This progressive reduction in the learning rate strategy enhances the stability and accuracy of the model training process. We employed cross-entropy loss as the loss function, and utilized the Adam optimizer^35^.

## Experimental results

In this study, we selected the image size that produced the best KL grade classification performance for each deep learning-based classification model: ResNet-101, ResNext-50–32×4d, Wide-ResNet-50-2, DenseNet-161, EfficientNet-b5, EfficientNet-V2-s, RegNet-Y-8GF, and ShuffleNet-V2-×2-0. The selected optimal image size is referred to as the *optimized image size*, and the weighted model trained with the optimized image size that performs the best is referred to as the *optimized model*. Next, we build an ensemble network by selecting the optimized model for each deep learning architecture. In addition, the image size of 224 × 224 is referred to as the *base image size*, and the weighted model trained with the 224 × 224 image size that performs the best is referred to as the *base model* for each deep learning architecture.

We selected the optimized image size and optimized model for each deep learning architecture for the KL grade classification task based on two performance metrics: classification accuracy (Accuracy) and F1-score. Accuracy is the evaluation metric that represents the KL grade classification performance of the models most intuitively. It is the ratio of the number of cases with the same predicted class and actual class to the total number of test data. However, Accuracy is not an objective performance metric when the deviation of the number of images per class in the dataset is large. To compensate for this issue, we also report the F1-score, which is an objective performance metric that is widely used when there is a severe imbalance among the classes in the dataset^36^. By amalgamating precision and recall into a single metric, the F1-score provides a more equitable assessment of model performance. It penalizes subpar predictions for minority classes while fostering improved generalization across the entire dataset. As indicated in Table 1, the distribution of training and test samples by KL grade exhibits a significant imbalance, with a ratio of grade 0: grade 1: grade 2: grade 3: grade 4 = 0.39:0.18:0.27:0.13:0.03. Given this pronounced class imbalance, the F1-score serves as a robust measure for evaluating model performance, particularly in situations where accurate classification of minority classes, such as grade 3 and grade 4, holds paramount importance.

Additionally, given the class imbalance observed among different KL grades within the dataset, traditional K-fold cross-validation is inadequate for precise performance evaluation. Consequently, in this study, we utilized the stratified fivefold cross-validation for both model training and performance assessment. The final output is the average of the five evaluations.

Table 2 shows the formulas for the performance metrics we used. TP (True Positive) and TN (True Negative) are outcomes where the predicted values match the actual value, and FP (False Positive) and FN (False Negative) are outcomes where the predicted values do not match the actual values. Among these, a TP is an outcome where the category of interest is accurately classified, whereas a TN is an outcome where the category that is not of interest is accurately classified. FP is an outcome where the category that is not of interest is incorrectly classified as the category of interest, and FN is an outcome where the category of interest is incorrectly classified as the category that is not of interest.

**Table 2:** Performance metrics equations.

Performance metric	Precision (PR)	Recall (RE)	F1-score	Accuracy
Formula	$\frac{{\text{TP}}}{{\text{TP}}+{\text{FP}}}$	$\frac{{\text{TP}}}{{\text{TP}}+{\text{FN}}}$	$2\times \frac{{\text{PR}}\times {\text{RE}}}{{\text{PR}}+{\text{RE}}}$	$\frac{{\text{TP}}+{\text{TN}}}{{\text{TP}}+{\text{FN}}+{\text{FP}}+{\text{TN}}}$

**Figure 4:**
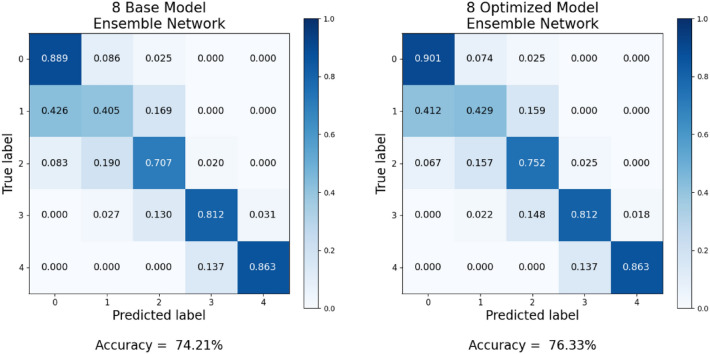
Confusion matrices of the eight-model ensemble networks. The optimized model ensemble network outperformed the base model ensemble in all grades. However, both types of ensemble networks exhibited a relatively lower prediction accuracy for KL grade 1 compared with that of the other grades.

Table 3 illustrates the KL grade prediction performance of each deep learning architecture and image size, as assessed through stratified fivefold cross-validation. Furthermore, Table 4 provides the average training time per epoch and model complexity for each model corresponding to its respective image size. As shown in Table 3, there is a significant performance difference depending on the image size. In the case of non-squared images, using images with a longer width than height consistently resulted in superior performance compared to that of the opposite cases. These findings are attributed to the increased distortion of joint space narrowing, a critical feature in KL grade assessment, when the height becomes proportionally longer. In most models, using square images showed better performance compared to using non-square images. When using non-square images, the RegNet-Y-8GF showed a slight improvement in accuracy, and the Wide-ResNet-50-2 model exhibited a marginal improvement in F1-score compared to their respective best performance with the use of square images. However, the improvement was minimal. Overall, the experimental results showed that the deep learning models provided the best or equivalent performance in KL grade assessment when using square images. Accordingly, the optimized image size was selected from square image sizes for each deep learning architecture. Subsequently, two ensemble networks (the base model ensemble network and optimized model ensemble network) were constructed using the selected base models and optimized models, respectively, and their Accuracy and F1-score were compared. Table 5 presents the KL grade classification performance results of the ensemble networks composed of eight models. Figure 4 presents the confusion matrices for the ensemble networks. The ensemble network consisting of the eight base models achieved an Accuracy of 74.21% and an F1-score of 0.7435, and the ensemble network consisting of the eight optimized models achieved an Accuracy of 76.33% and an F1-score of 0.7640. When the ensemble network was constructed using the optimized models instead of the base models, both Accuracy and F1-score increased. In the case of the optimized model ensemble network, there is a noticeable improvement in the prediction accuracy for each KL grade, and the performance of the individual deep learning models that make up the ensemble has also been enhanced. According to the experimental results, prediction accuracy for KL grade 1 is consistently lower than that for other grades in all cases. Interestingly, even when employing the base models and the ensemble network built upon them, the classification performance for grades 3 and 4, representing the minority class, remains high. Furthermore, it was observed that the performance further improved with the optimized models corresponding to these base deep learning models. These findings align with the analysis using the Precision–Recall (PR) curve, which will be presented shortly.

**Table 3:** KL grade classification performance of different models according to image size. In the case of non-squared images, using images with a longer width than height consistently resulted in superior performance compared with that of the opposite cases. However, in most models, using square images showed better performance compared with using non-square images.

Model	DenseNet-161	EfficienNet-b5	EfficienNet-V2-s	RegNet-Y-8GF	ResNet-101	ResNext-50-32×4d	Wide-ResNet-50-2	ShuffleNet-V2-×2-0
Image size	Accuracy (F1-score)							
224 × 224	0.6999 (0.6921)	0.7105 (0.6992)	0.7233 (0.7214)	0.7209 (0.7307)	0.7023 (0.7048)	0.7122 (0.7066)	0.7023 (0.6947)	0.6909 (0.6884)
300 × 300	0.6935 (0.6777)	0.7031 (0.6941)	0.7324 (0.7203)	0.7297 (0.7240)	0.7120 (0.7090)	0.7170 (0.7104)	0.7122 (0.7081)	0.7075 (0.6988)
384 × 384	0.7041 (0.7057)	0.7222 (0.7236)	0.7379 (0.7299)	0.7373 (0.7340)	0.7227 (0.7115)	0.7228 (0.7141)	0.7112 (0.7148)	0.7152 (0.7094)
448 × 448	0.7133 (0.7040)	0.7361 (0.7329)	0.7362 (0.7365)	0.7372 (0.7339)	0.7190 (0.7152)	0.7214 (0.7158)	0.7180 (0.7114)	0.7143 (0.7113)
456 × 456	0.7121 (0.7083)	0.7306 (0.7289)	0.7442 (0.7326)	0.7353 (0.7362)	0.7118 (0.7136)	0.7233 (0.7122)	0.7208 (0.7125)	0.7110 (0.7076)
512 × 512	0.7156 (0.7045)	0.7355 (0.7301)	0.7406 (0.7415)	0.7271 (0.7313)	0.7152 (0.7105)	0.7261 (0.7206)	0.7081 (0.7094)	0.7161 (0.7164)
224 × 336 (1: 1.5)	0.6860 (0.6763)	0.7080 (0.7082)	0.7246 (0.7195)	0.7207 (0.7187)	0.7062 (0.7012)	0.7081 (0.7034)	0.7086 (0.6953)	0.6988 (0.6872)
336 × 224 (1.5: 1)	0.6963 (0.6821)	0.7088 (0.7151)	0.7289 (0.7234)	0.7329 (0.7218)	0.7176 (0.7050)	0.7140 (0.7060)	0.7087 (0.7054)	0.7017 (0.6946)
224 × 448 (1:2)	0.6957 (0.6863)	0.7100 (0.7023)	0.7194 (0.7241)	0.7220 (0.7176)	0.6970 (0.7013)	0.7128 (0.7053)	0.7001 (0.6898)	0.6897 (0.7026)
448 × 224 (2:1)	0.6953 (0.6883)	0.7190 (0.7142)	0.7245 (0.7230)	0.7390 (0.7333)	0.7156 (0.7122)	0.7245 (0.7135)	0.7028 (0.6970)	0.7130 (0.7064)
300 × 450 (1:1.5)	0.6999 (0.6907)	0.7155 (0.7186)	0.7366 (0.7292)	0.7297 (0.7225)	0.7074 (0.7076)	0.7120 (0.7102)	0.7103 (0.7016)	0.6960 (0.6959)
450 × 300 (1.5:1)	0.7017 0.7032)	0.7280 (0.7262)	0.7364 (0.7368)	0.7343 (0.7309)	0.7103 (0.7077)	0.7237 (0.7138)	0.7157 (0.7145)	0.6988 (0.7057)

**Table 4:** Average training time per epoch and model complexity.

	224 × 224	300 × 300	384 × 384	448 × 448	456 × 456	512 × 512	224 × 336	336 × 224	224 × 448	448 × 224	300 × 450	450 × 300	Number of parameters
DenseNet-161	2 m 4 s	2 m 15 s	2 m 33 s	2 m 50 s	2 m 51 s	3 m	2 m 26 s	2 m 27 s	2 m 29 s	2 m 26 s	2 m 35 s	2 m 38 s	28.7 M
EfficientNet-b5	1 m 59 s	2 m 5 s	2 m 22 s	2 m 28 s	2 m 32 s	2 m 41 s	2 m 12 s	2 m 14 s	2 m 17 s	2 m 17 s	2 m 29 s	2 m 27 s	30.4 M
EfficientNet-V2-s	1 m 50 s	1 m 53 s	2 m 9 s	2 m 13 s	2 m 19 s	2 m 22 s	2 m 1 s	2 m 5 s	2 m 7 s	2 m 9 s	2 m 15 s	2 m 16 s	21.5 M
RegNet-Y-8GF	1 m 17 s	1 m 38 s	2 m 18 s	3 m 5 s	3 m 11 s	3 m 46 s	1 m 42 s	1 m 39 s	1 m 56 s	1 m 56 s	2 m 19 s	2 m 18 s	39.4 M
ResNet-101	1 m 11 s	1 m 15 s	1 m 39 s	1 m 49 s	1 m 50 s	1 m 59 s	1 m 27 s	1 m 26 s	1 m 31 s	1 m 33 s	1 m 43 s	1 m 43 s	44.5 M
ResNext-50-32×4d	53 s	1 m 1 s	1 m 19 s	1 m 30 s	1 m 33 s	1 m 42 s	1 m 5 s	1 m 9 s	1 m 14 s	1 m 15 s	1 m 26 s	1 m 28 s	25.0 M
Wide-ResNet-50-2	52 s	1 m 4 s	1 m 24 s	1 m 40 s	1 m 42 s	1 m 54 s	1 m 9 s	1 m 9 s	1 m 19 s	1 m 21 s	1 m 33 s	1 m 33 s	68.9 M
ShuffleNet-V2-×2-0	46 s	59 s	1 m 16 s	1 m 20 s	1 m 21 s	1 m 24 s	1 m 3 s	1 m 3 s	1 m 10 s	1 m 10 s	1 m 20 s	1 m 21 s	7.4 M

**Table 5:** KL grade classification performance of the eight-model ensemble network. When the ensemble network was constructed using the optimized models instead of the base models, both Accuracy and F1-score increased.

Eight-model ensemble network						
	Base model ensemble network			Optimized model ensemble network		
KL grade	Precision	Recall	F1	Precision	Recall	F1-score
0	0.7770	0.8889	0.8292	0.7912	0.9014	0.8427
1	0.4511	0.4054	0.4270	0.5100	0.4291	0.4661
2	0.7689	0.7069	0.7366	0.7778	0.7517	0.7645
3	0.9188	0.8117	0.8619	0.9095	0.8117	0.8578
4	0.8627	0.8627	0.8627	0.9167	0.8627	0.8889
Mean	0.7557	0.7351	0.7435	0.7810	0.7513	0.7640
Accuracy	0.7421			0.7633

**Figure 5:**
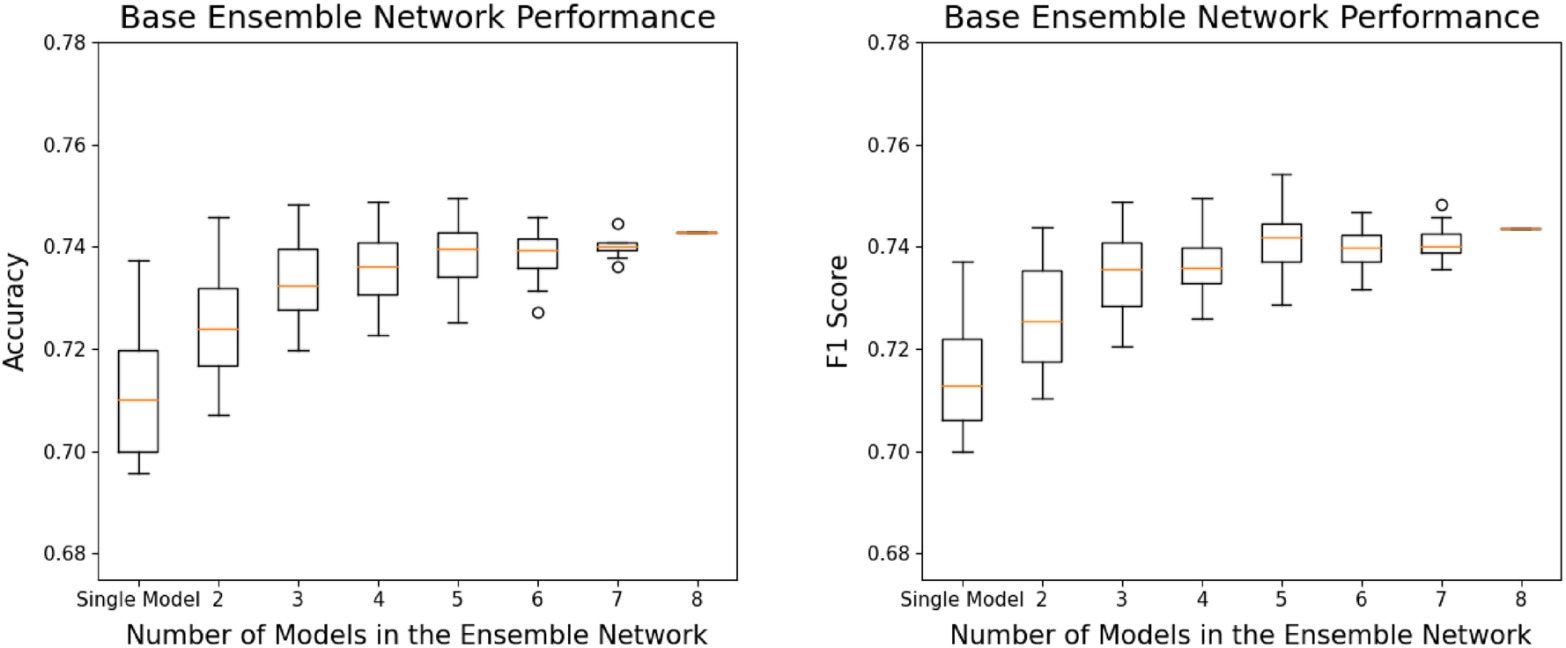
Base model ensemble network performance. The average performance improves as the number of models in the ensemble network increases. However, performance improvement plateaus as the number of models reaches a certain point, and the best performance is achieved with five base models.

**Figure 6:**
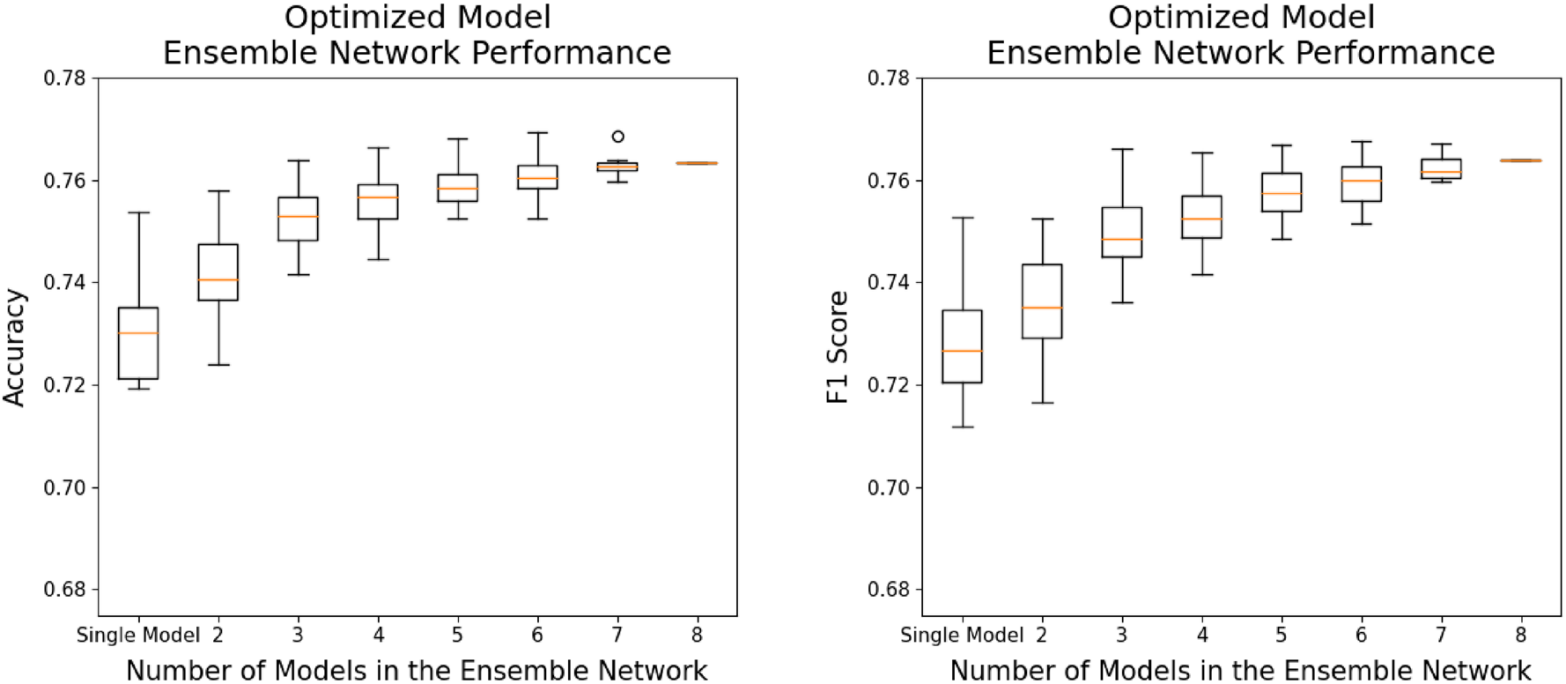
Optimized model ensemble network performance. Similar to the case of the base model ensemble network, the average accuracy and F1-score improve as the number of models in the ensemble network increases, and the ensemble network with six optimized models exhibited the best performance.

It is known that the number of classification models comprising an ensemble network has a significant effect on the prediction performance^37^. To investigate this, we conducted a performance comparison among all possible combinations of ensemble networks with different component networks and numbers of classification models. In particular, to evaluate the number of classification models comprising the ensemble networks, we conducted performance experiments on all combinations that could be configured with that number of classification models and the voting algorithms (e.g., a soft voting ensemble and mix voting ensemble). Among these, the performance of the ensemble network that performed the best was identified as the representative performance for that number of classification models. Box plots are used in Figs. 5 and 6 to present the performance of the base model and optimized model ensemble networks, respectively.

According to Figs. 5 and 6, the optimized model ensemble network generally outperforms the base model ensemble network in terms of KL grade prediction Accuracy and F1-score. However, the average performance improves, and the deviation in performance decreases for both ensemble networks as the number of models comprising the ensemble network increases. These results reconfirm the findings from previous research that ensemble networks can overcome the instability of single models and improve prediction ability.

**Figure 7:**
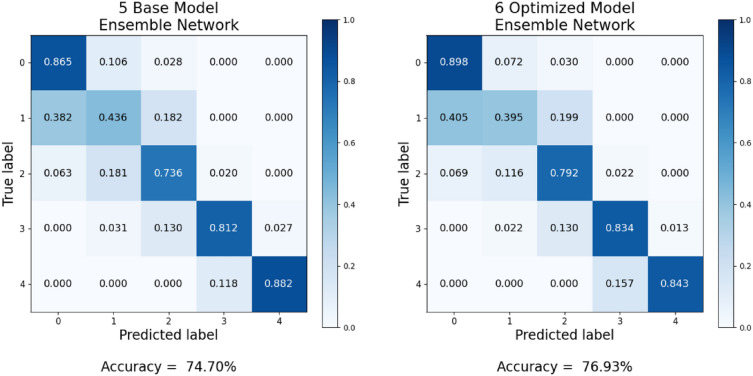
Confusion matrices of the best-performing ensemble networks. Overall, the ensemble network with six optimized models outperformed the one with fixed base models in terms of prediction accuracy. However, both models exhibited relatively lower predictive performance for KL grade 1 compared with the performance of other grades, and the prediction accuracy of the six optimized model ensemble network (Accuracy = 0.395) was slightly lower than that of the five base model ensemble network (Accuracy = 0.436).

The average Accuracy and F1-score improve for both ensemble networks as the number of models comprising the ensemble network increases. However, performance improvement plateaus when the number of models reaches a certain number. For example, in the case of the optimized model ensemble network, average Accuracy and F1-score plateau when the ensemble network consists of five or more models. The same phenomenon is also observed for the base model ensemble network. Furthermore, a similar phenomenon occurs when peak performance is considered. For example, the ensemble network consisting of five base models (EfficientNet-V2-s, RegNet-Y-8GF, ResNext-50-32×4d, Wide-ResNet-50-2, ShuffleNet-V2-×2-0) achieved the best performance among the base model ensemble networks. Among the optimized model ensemble networks, the ensemble network consisting of six optimized models (EfficientNet-b5, EfficientNet-V2-s, RegNet-Y-8GF, ResNet-101, ResNext-50-32×4d, ShuffleNet-V2-×2-0) achieved the best performance. Table 6 presents the KL grade classification performance for the base and optimized model ensemble networks with the best performance, and Fig. 7 shows the confusion matrices of these ensemble networks.

**Table 6:** KL grade classification performance of the best-performing ensemble networks. The best-performing base model ensemble network consists of five base models, while the best-performing optimized model ensemble network comprises six optimized models. The optimized model ensemble network exhibited superior performance across all KL grades.

Best-performing ensemble network						
	Base model ensemble network			Optimized model ensemble network		
Constituting models	EfficientNet-V2-s, RegNet-Y-8GF, ResNext-50-32×4d, Wide-ResNet-50-2, ShuffleNet-V2-×2-0			EfficientNet-b5, EfficientNet-V2-s, RegNet-Y-8GF, ResNet-101, ResNext-50-32×4d, ShuffleNet-V2-×2-0		
KL grade	Precision	Recall	F1	Precision	Recall	F1-score
0	0.7968	0.8654	0.8297	0.7917	0.8983	0.8416
1	0.4526	0.4358	0.4441	0.5318	0.3953	0.4535
2	0.7651	0.7360	0.7503	0.7679	0.7919	0.7797
3	0.9235	0.8117	0.8640	0.9118	0.8341	0.8712
4	0.8824	0.8824	0.8824	0.9348	0.8431	0.8866
Mean	0.7641	0.7463	0.7541	0.7876	0.7525	0.7665
Accuracy	0.7470			0.7693

For the ensemble networks that exhibited the best performance, we investigated the impact of different voting algorithms (see Table 7). In both the base model and optimized model ensemble networks, the mix voting algorithm demonstrated a slight improvement in performance compared to other voting algorithms, such as hard and soft voting algorithms. The reason behind these results is straightforward. The mix and hard voting algorithms are essentially the same, with the sole difference being that ties are resolved using the soft voting algorithm. Therefore, the performance gap among these voting algorithms can diminish when there are fewer test images with equal votes or when most individual component deep learning models exhibit good performance. For the base models, the ensemble network with mix voting showed an improvement in Accuracy from 0.65 to 6.67% and an enhancement in F1-score from 0.87 to 6.21%. Similar performance improvements were also observed in the optimized models. The optimized model ensemble network using mix voting outperformed individual optimized models in Accuracy, showing an improvement from 2.08 to 6.61%, and in F1-score, with an increase from 1.82 to 6.21%.

**Table 7:** Performance of individual deep learning models comprising best-performing ensemble networks. In both the base model and optimized model ensemble networks, the mix voting algorithm demonstrated a slight improvement in performance compared with that of other voting algorithms.

Model	DenseNet-161	EfficientNet-b5	EfficientNet-V2-s	RegNet-Y-8GF	ResNet-101	ResNext-50-32×4d	Wide-ResNet-50-2	ShuffleNet-V2-×2-0	Hard voting	Soft voting	Mix voting
	Accuracy (F1-score)										
Base model ensemble network (5 models)	Not used	Not used	0.7373 (0.7322)	0.7216 (0.7371)	Not used	0.7192 (0.7186)	0.7011 (0.7095)	0.6957 (0.7000)	0.7349 (0.7363)	0.7428 (0.7414)	0.7421 (0.7435)
Optimized model ensemble network (6 models)	Not used	0.7337 (0.7292)	0.7536 (0.7528)	0.7373 (0.7399)	0.7216 (0.7217)	0.7343 (0.7330)	Not used	0.7264 (0.7171)	0.7585 (0.7566)	0.7639 (0.7634)	0.7693 (0.7665)

While the optimized model ensemble networks outperformed the base model ensembles, both types of ensemble networks exhibited notably lower prediction accuracy for KL grade 1 when compared to the other grades (see Figs. 4, 7). We conjecture that these results may be attributed to the possibility that clear distinguishing features between grade 0 and grade 1 may not be easily discernible in the radiographs, resulting in incorrect classifications. For instance, KL grade 1 indicates subtle changes from KL grade 0, such as mild joint space narrowing or the potential presence of small osteophytes, which can introduce ambiguity into the classification^11^. This observation aligns with the findings of prior studies, including those by Chen et al.^2^, Yong et al.^11^, Tiulpin et al.^14^, and Jain et al.^17^, all of which yielded a considerably lower rate of correct predictions for KL grade 1 images when compared to the other grades.

**Figure 8:**
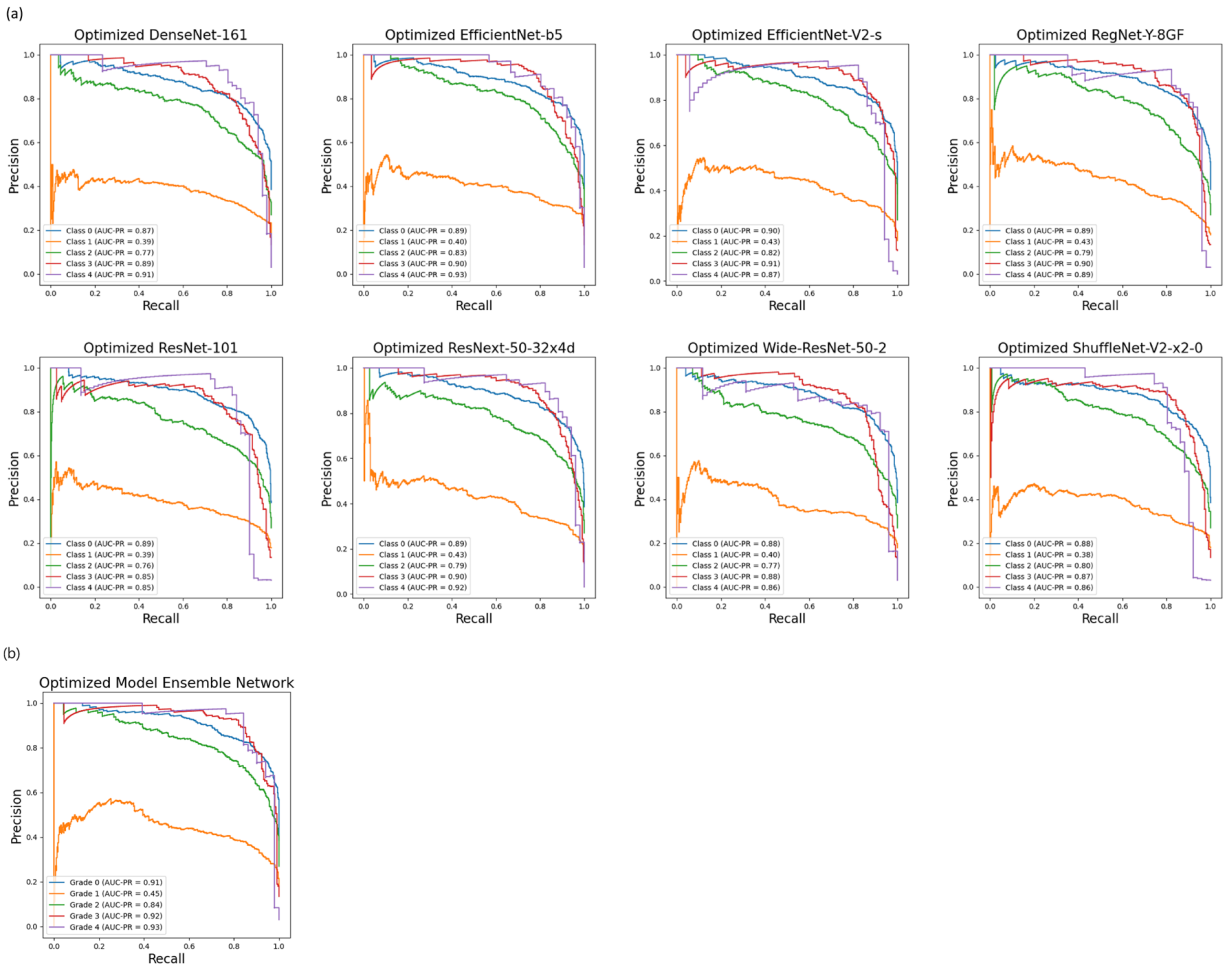
Precision–Recall curves for: (**a**) the eight deep learning models trained using individual optimal image size, and (**b**) the optimized eight-model ensemble network.

To analyze the performance of the optimized model ensemble network and its constituent deep learning models in the KL grade classification task, considering the presence of class imbalance, we generated corresponding Precision–Recall (PR) curves (see Fig. 8). All individual deep learning models exhibited high Area Under the PR curve (AUC-PR) values for class 3 and class 4, despite the relatively limited amount of training data. Furthermore, within the ensemble network composed of these models, the AUC-PR values demonstrated additional improvement (e.g., KL grade 3: 0.92, KL grade 4: 0.93). This reaffirms that training with the optimal image size tailored to each deep learning model facilitates more effective feature learning, even when dealing with a constrained amount of training data, as opposed to training with a uniform image size.

Regarding the class imbalance within the OAI dataset, several previous studies^2,11,14,17^ have reported analogous findings. Specifically, in the absence of dedicated sampling strategies, deep learning models demonstrated robust prediction accuracy for the minority classes, specifically KL grade 3 and KL grade 4. Conversely, across all these studies, prediction accuracy for KL grade 1, which benefits from a relatively larger training dataset than the minority classes, exhibited a notable decline. However, it remains uncertain whether the techniques and algorithms employed in these studies will yield equivalent results when applied to actual clinical data. Furthermore, their effectiveness in medical image analysis for different diseases or conditions cannot be guaranteed. Hence, we advocate for more comprehensive experiments and analyses concerning class imbalances, contingent on the specific medical data in use.

**Figure 9:**
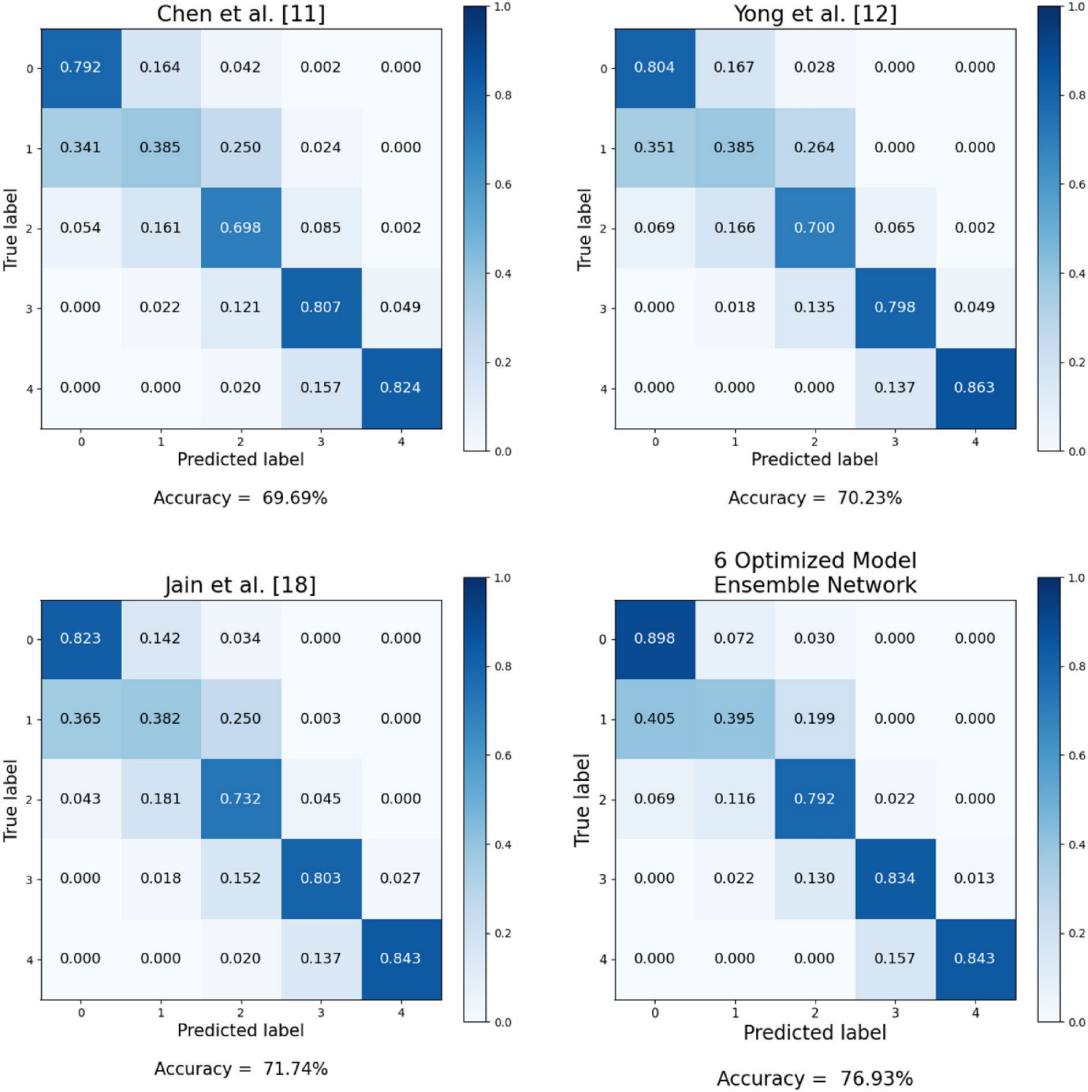
Confusion matrices for the KL grades predicted using the proposed methods and existing work. The proposed method demonstrated the highest performance with an accuracy of 76.93%. However, all methods exhibited a significantly lower prediction accuracy for KL grade 2 compared with that of the other grades.

Table 8 and Fig. 9 compare the performance results of the methods proposed in this study with those of previous studies. The method proposed in this paper outperforms existing techniques that have shown outstanding performance in KL grade classification. In particular, the Accuracy is improved by about 5.19%, and the F1-score is improved by about 0.5 when compared with the results of OsteoHRNet, which is the most recently published technique^18^. However, it was observed that all methods exhibited a significantly lower prediction accuracy for KL grade 2 compared with that of the other grades.

**Table 8:** Comparison between KL grade classification performance of the proposed method and that of previous studies.

Method	Accuracy (%)	F1-score
VGG-19 + Ordinal^11^	69.69	0.70
DenseNet-161 + ORM^12^	70.23	0.70
OsteoHRNet^18^	71.74	0.72
Ours (Optimized model network ensemble with 6 models)	76.93	0.77

### Model interpretation

**Figure 10:**
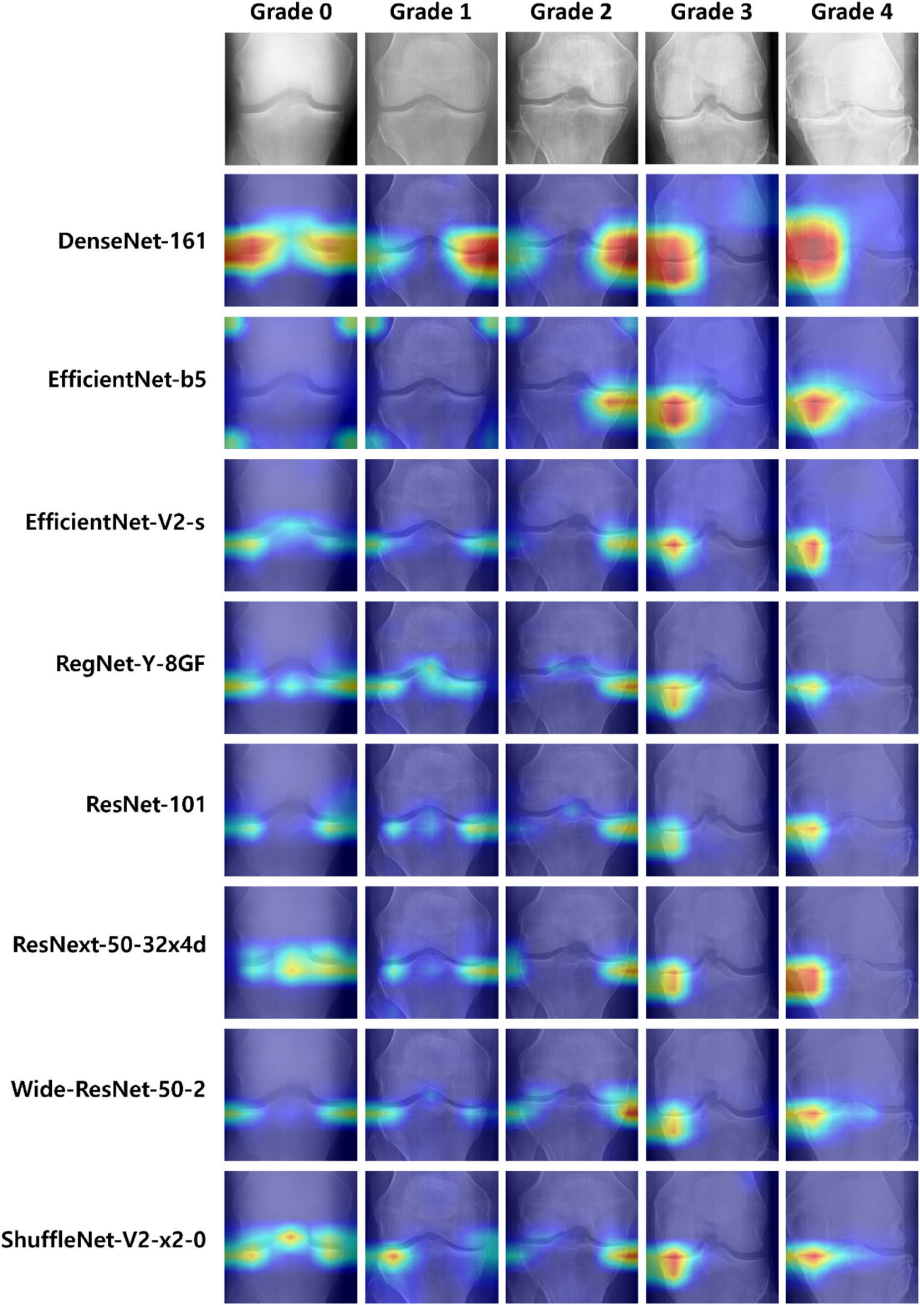
Grad-CAM results for each base model trained with the base image size (224 × 224).

**Figure 11:**
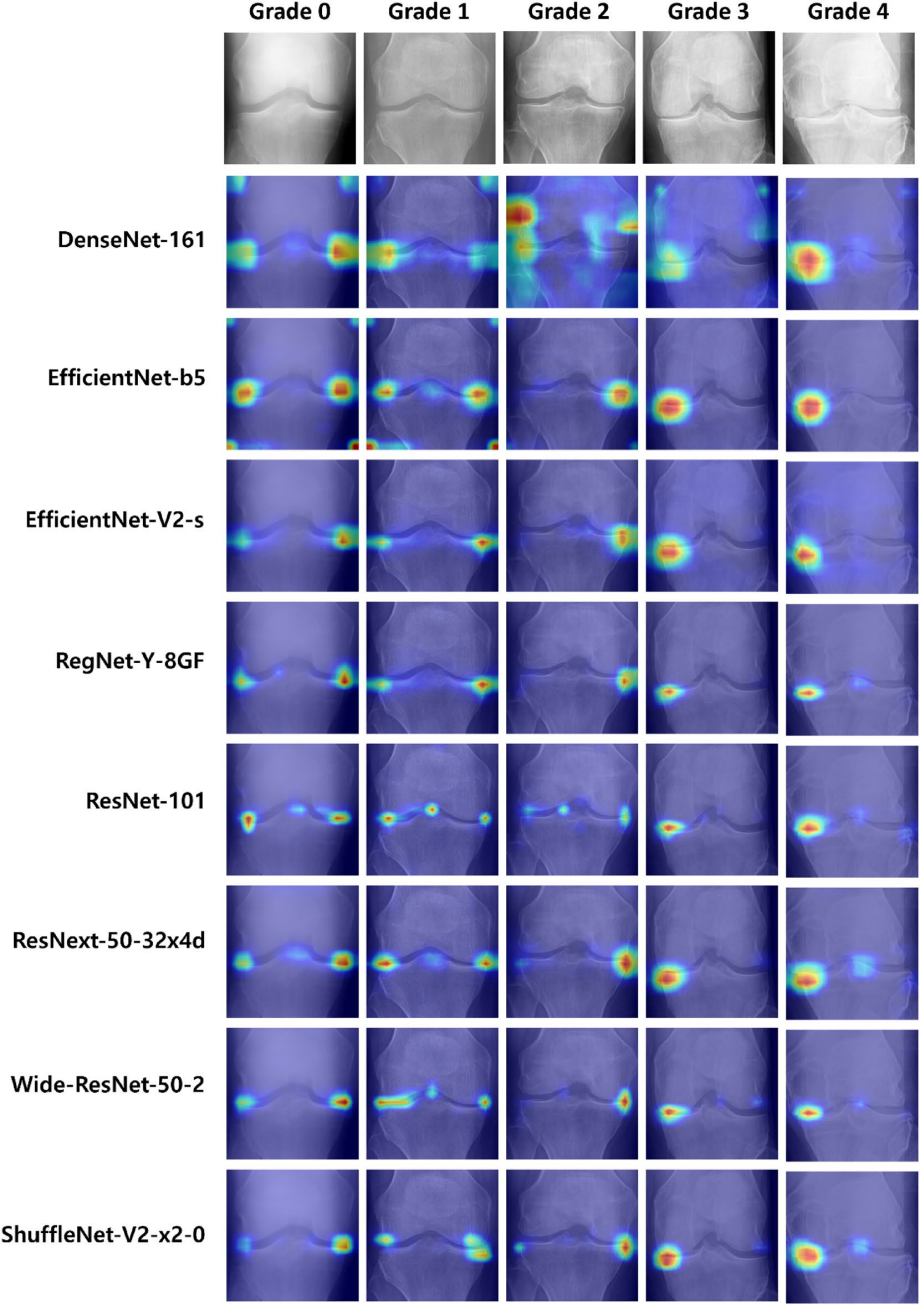
Grad-CAM results for each optimized model trained with the optimized image size. Compared with the base models, the individual optimized models better focused on the area around the knee joint to extract the important features required for KL grade classification.

In this study, we used the Grad-CAM visualization technique^38^ to visually represent the area of the image that receives the most attention when each of the eight models classifies the KL grade. As a result, we can determine whether each model classifies the KL grade correctly according to the features of the KL grades. For example, in Fig. 10, we can determine the Grad-CAM results of the individual base models for the knee X-ray images of each KL grade. Figure 11 shows the Grad-CAM results for the individual optimized models for the knee X-ray images of each KL grade.

**Figure 12:**
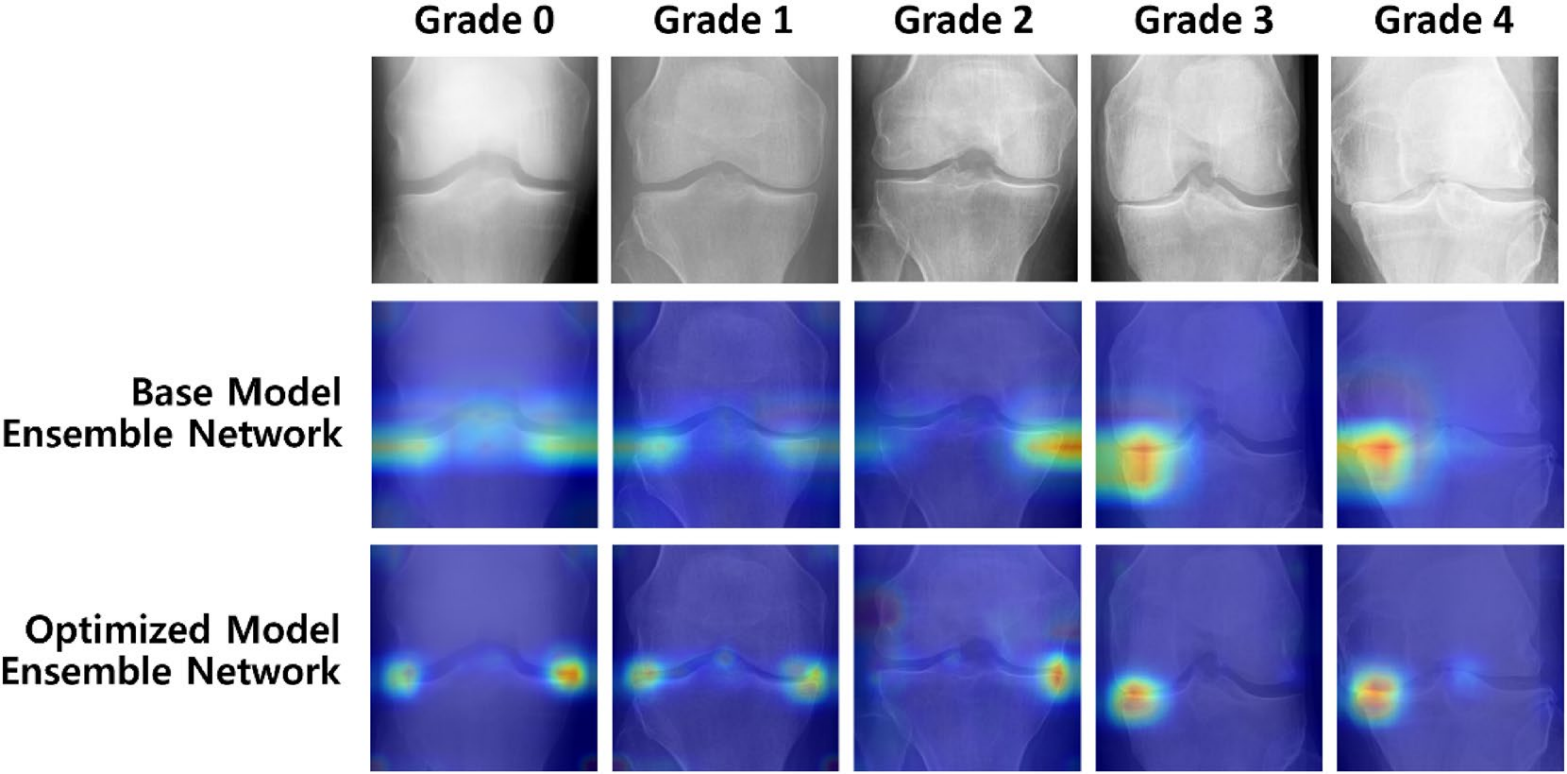
Ensemble Grad-CAM of the base and optimized model ensemble networks for different KL grades. The deviation of the focused areas is significantly reduced in the optimized model ensemble network.

Figure 12 shows the ensemble Grad-CAM results for the base and optimized model ensemble networks consisting of eight classification models. Each pixel value of the ensemble Grad-CAM images is calculated as the average of the pixel values of the Grad-CAMs generated by each of the eight classification models comprising the corresponding ensemble network. Unlike the base models, the optimized models and optimized model ensemble network focus on the area surrounding the joint space of the knee to extract imaging features such as joint space narrowing, the formation of osteophyte, and osteosclerosis, which are needed to classify the KL grade, as shown in the experimental results. Furthermore, when comparing the Grad-CAM results of each optimized model and that of the ensemble network to identify the areas of the knee X-ray images that are focused on when classifying the KL grade, the ensemble Grad-CAM shows that the deviation is significantly reduced in the ensemble network.

## Conclusion

In this study, we constructed an ensemble network using conventional and representative image classification models for images of natural scenes to predict the KL grade for knee OA. In addition, we analyzed the performance of the ensemble network through various experiments. In particular, we did not use a dataset with a single image size. Instead, to train the models, we used a knee X-ray dataset with the optimized image sizes—that is, we used the image size most suitable for training each model comprising the ensemble network. We then used the trained models to construct the ensemble network. In addition, we demonstrated through experiments that this ensemble-based method not only improves the classification accuracy of each model but also overcomes the instability of a single model and further enhances the prediction capability. Furthermore, we verified using the Grad-CAM visualization technique that the optimized models trained with the optimized image size and the optimized model ensemble network consisting of the optimized models correctly learned the features of each KL grade in the knee X-ray images and inferred the correct KL grade.

Our future research plan is outlined as follows. First, in this study, because the original image size was 224 × 224, maintaining the aspect ratio while resizing the image can be considered to improve performance, as it prevents any distortions of the bone’s shape compared to resizing it into a non-square image. However, in cases where the original image itself is not square, resizing it into a square does not always guarantee superior performance; therefore, further research is needed on such situations. Second, although the proposed model training and ensemble technique, utilizing the optimized image size, have demonstrated superior performance compared to existing deep learning-based solutions, it is noteworthy that the proposed ensemble network frequently misclassifies KL grade 1 as KL grade 0 or KL grade 2. This issue can be attributed to the limitations of the deep learning models in capturing very subtle features present in KL grade 1 images. Therefore, we intend to conduct further research to address this challenge. Third, the training and validation in this study were performed using publicly available datasets. In our future work, we plan to incorporate external datasets from multiple institutions that are entirely distinct from those used in this study. This will enable us to assess the generalization performance of the proposed method on a broader scale. Finally, we aim to enhance the KL grade classification model by employing a wider range of deep learning-based classification models and conducting hyperparameter optimization to achieve improved performance.

## Data Availability

The dataset used in this study is publicly accessible at https://data.mendeley.com/datasets/56rmx5bjcr/1 (accessed on May 30, 2023).

## Code availability

The source code developed in this study are available at https://github.com/RTOS-KGU/KLGrade.
